# Overview of tobacco cessation service in Oman: A narrative review

**DOI:** 10.18332/tpc/201992

**Published:** 2025-03-27

**Authors:** Salma R. Al-Kalbani

**Affiliations:** 1 Directorate General of Health Services, Muscat, Oman

**Keywords:** tobacco cessation, Oman, public health

## Abstract

Tobacco is a global public health issue that kills half of its users. Even though the Framework Convention on Tobacco Control (FCTC) mandates countries to implement tobacco cessation programs as a cost-effective approach to assist smokers in quitting and reduce the burden of tobacco use, only one-third of the world's population has access to effective cessation services. Many governments have failed to provide comprehensive, accessible tobacco cessation services due to financial constraints and the belief that people are to blame for tobacco addiction. The World Health Organization's Package of Essential Non-Communicable Diseases (WHO PEN) recommends incorporating tobacco cessation and lifestyle advice into primary healthcare as a cost-effective means of avoiding non-communicable diseases. Despite nearly two decades have passed since ratifying the Framework Convention on Tobacco Control (FCTC), Oman has made little progress in developing a national comprehensive tobacco cessation program. A comprehensive multisectoral effort is necessary to explore the challenges and opportunities for implementing an effective national tobacco cessation program in Oman, which should be part of effective tobacco control legislation aimed at reversing the tobacco trend and assisting smokers in quitting tobacco products. This narrative review aims to explore tobacco use in Oman, its health impacts, strengths, weaknesses, opportunities, and threats, and puts forward recommendations for implementing a tobacco cessation program.

## INTRODUCTION

Tobacco use is a global public health issue that kills half of its users, with effects being unevenly distributed among and within countries^1^. Tobacco use increases the risk of cardiovascular and respiratory disorders, more than 20 different types or subtypes of cancer, unfavorable pregnancy outcomes, and a variety of other chronic health conditions^1^. The Framework Convention on Tobacco Control (FCTC), which aims to reduce the burden of tobacco use, mandates countries to disseminate guidelines on tobacco cessation and promote effective treatment^2^. However, only one-third of the world’s population has access to effective cessation services^3^. Many governments have failed to provide a comprehensive, accessible tobacco cessation service due to financial constraints and the belief that people are responsible for tobacco addiction. Failure to offer and promote cessation support to tobacco users is not only a public health failure but also a human rights failure^2^.

Tobacco control is an integral part of the Sustainable Development Goals (SDG) as stated in Goal 3.a, as well as achieving the direct and indirect impacts of curbing tobacco use^4^. The current tobacco epidemic should be seen as a public health emergency that cannot be effectively addressed without a comprehensive tobacco cessation program^2^. The WHO’s Package of Essential Non-Communicable Diseases (WHO PEN) suggests integrating tobacco cessation and lifestyle counseling within primary healthcare as a cost-effective method of preventing non-communicable diseases^5^. The revised global non-communicable disease action plan 2013–2020 suggests three population-wide cessation techniques as cost-effective interventions to reduce tobacco prevalence: a nationwide toll-free quitline, mCessation (mobile phone-based intervention offering text messages to assist individuals in their efforts to quit smoking) and brief advice^6^. These measures should be implemented in conjunction with effective cessation services that offer behavioral and pharmacological treatment to tobacco users. Tobacco cessation services are most effective when combined with other tobacco control policies, such as raising tobacco taxes, promoting smoke-free environments, banning tobacco advertising, promotion, and sponsorship, implementing plain packaging, and delivering anti-tobacco mass media campaigns^2^. These collective measures promote quitting and foster a supportive environment. Thus, countries that ratify the WHO FCTC are obligated to design a comprehensive national tobacco cessation program as part of an effective multisectoral tobacco control initiative and implement it in a way that provides accessible and affordable cessation support^2^.

Oman joined the WHO FCTC in 2005 as part of a global initiative to combat the tobacco epidemic^2^. Currently, there is a subnational tobacco control office that has representatives from different sectors^7^. However, despite that two decades have passed since ratifying the FCTC, Oman is yet to achieve the best practice approach in all tobacco control measures, including general obligations, demand reduction measures, and supply reduction measures^8^. Specifically, tobacco taxes in Oman are less than 75% of the total retail price^9^. There is no 100% smoke-free environment in enclosed public places, as some exemptions are allowed^10^. Tobacco advertising, promotion, and sponsorship are strictly prohibited, but some are permitted under the banner of corporate social responsibility^10^. Neither a comprehensive tobacco cessation program nor a nationwide mass media campaign to increase public awareness of tobacco products exists^10^. As of April 2024, plain packaging for tobacco products came into effect which seeks to establish an Omani Standard Specification Binding^11^. The youngest legal age for tobacco use is 18 years, but evidence suggests initiation occurs at younger ages^12^, exacerbated by inadequate data on illicit tobacco trade, particularly for smokeless and innovative tobacco products^12^. This is reflected by increase in the prevalence of tobacco use in Oman^13^. Even though the 2040 national health goal is to create a healthy society free of health risks and hazards, evidence on incorporating tobacco control measures into national health strategies or other national strategies, is limited^14^. The biggest challenge is the absence of comprehensive national tobacco control legislation that aims to ensure the effective implementation and enforcement of different tobacco control measures in Oman^10^.

In Oman, little progress has been made in building a national tobacco cessation program that satisfies Article 14 of WHO FCTC at its best practice, which requires having a national toll-free Quitline, cessation counselling, and cessation medication^9^. Even though the national priority in Oman is to address, prevent, and control NCDs^15^, with a special emphasis on predisposing risk factors such as tobacco use, the healthcare system has several challenges in achieving its obligations under the WHO FCTC Article 14. The presence of a tobacco cessation program can ensure that smokers receive assisted tobacco cessation treatment and improve their chances of quitting successfully. Tobacco cessation programs work best as part of a nationwide comprehensive, multisectoral tobacco control initiative that aims to curb tobacco use and assist tobacco users in quit smoking^2^. However, the policymakers are still considering tobacco use as an individual problem for which the patient is to blame and ignoring the wider social and commercial determinants of health that affect individual choices. This is demonstrated in the absence of an effective tobacco cessation program at the national or subnational level. To the author’s knowledge, data that assess tobacco cessation service in Oman are limited; however, no formal assessment has been made to analyze the current situation of tobacco cessation service. This review will examine the epidemiological data on tobacco cessation in Oman, the impact of tobacco use, the strengths, weaknesses, opportunities and threats, to determine the window of opportunity and the path forward for developing a comprehensive tobacco cessation program.

## TOBACCO USE IN OMAN

### The increasing prevalence of tobacco use

**Figure 1:**
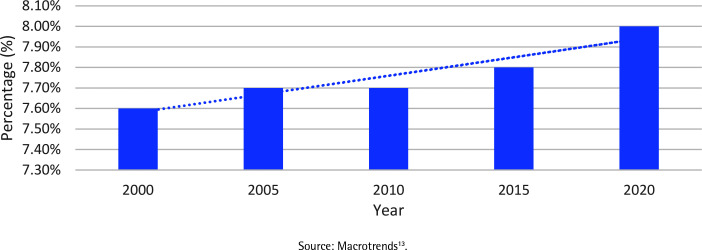
Age-standardized smoking prevalence among adults in Oman (aged ≥15 years)

The prevalence of tobacco consumption in Oman has increased in recent years, reaching 8.0% in 2020, raising concerns about its short- and long-term health consequences (Figure 1)^13^. According to the Global Burden of Disease (GBD) 2019, nearly 9% of all deaths in Oman were attributed to tobacco use^16^. Although Oman does not manufacture tobacco products, in 2022, Oman produced 1121 tons of tobacco on 249 hectares of fertile agricultural land that could have been used to grow food^8^. The increase in tobacco prevalence is most likely driven by the inadequate implementation and enforcement of a comprehensive tobacco control program that addresses demand and supply reduction measures, allowing the tobacco industry to thrive and use different tactics to sell their deadly products. Despite the fact that the World Health Assembly established a global goal of a 30% reduction in relevant tobacco prevalence by 2025 as part of the noncommunicable disease monitoring framework, available data indicated that Oman would not achieve this target^17^.

### Tobacco usage among adults

The estimated prevalence of current adult tobacco use, both smoked and smokeless tobacco products, in Oman in 2020 was 8.0%, with male prevalence being significantly higher than female prevalence (Table 1)^18^. Furthermore, although there is no prior data to compare with, the 2020 statistic revealed that 6.1% of adults in Oman smoked cigarettes. Although it is banned in Oman, the adult prevalence of current smokeless tobacco use is 1% (1.8% in males and 0.1% in females)^19^. No data are available about the prevalence and extent of innovative tobacco products, e.g. e-cigarettes and e-hookahs, among adults in Oman. Although it is emerging as an acceptable culture in Oman, the data on the prevalence of waterpipe (shisha) smoking in Oman are limited, as is the case on a global scale.

**Table 1:** Estimate of current tobacco smoking prevalence (%) in the last 30 days in Oman, 2000, 2010, and 2020 (age standardized rate)

*Variable*	*2000*			*2010*			*2020*		
	*Total*	*Male*	*Female*	*Total*	*Male*	*Female*	*Total*	*Male*	*Female*
**Current tobacco use**	7.6	14.5	0.7	7.7	14.9	0.5	8.0	15.5	0.4
**Current tobacco smoking**	6.9	13.2	0.6	7.0	13.6	0.4	7.2	14.0	0.3
**Current cigarette use**	NA	NA	NA	NA	NA	NA	6.1	12.0	0.1

Source: The Global Health Observatory^18^. NA: not available.

### Tobacco usage among children

**Figure 2:**
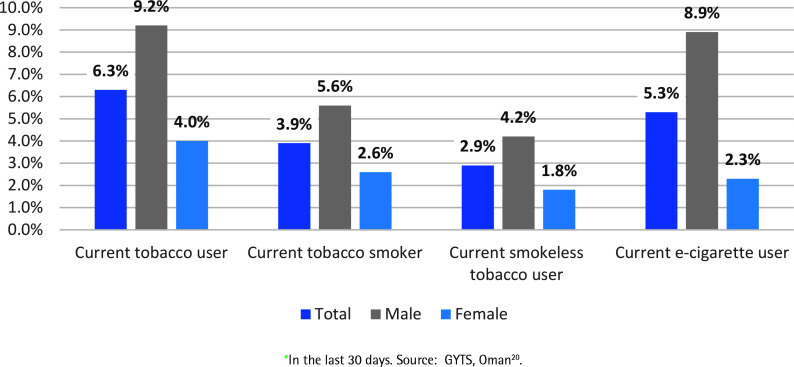
Current tobacco use* among students aged 13-15 years in Oman based on Global Youth Tobacco Survey , Oman 2016 (N=1498)

The Global Youth Tobacco Survey 2016 highlighted a worrying public health issue in tobacco smoking among children in Oman, given that these products are banned before the age of 18 years (Figure 2)^20^. According to the survey, 6.9% of children surveyed were current users of tobacco products, with prevalence among males double that of females (9.2% vs 4.0%, respectively). Current tobacco smokers accounted for 3.9%, while current users of smokeless tobacco products accounted for 2.9%. E-cigarette use accounted for 5.3%, with prevalence among boys higher than that of girls (8.9% vs 2.3%, respectively). According to Oman’s GYTS data, tobacco use is primarily reported to take place at homes, while schools also record tobacco usage. This raises issues about school policies that encourage healthy behaviors, as well as the influence of families on children’s smoking habits^20^. There are no nationally representative data on tobacco use among youth since 2016, which makes it difficult to measure the impact of the problem and subsequently manage it.

### Tobacco products are addictive

It has been established that nicotine is addictive, possibly much more than the addictions caused by other illegal substances^21^. The amount of nicotine present in tobacco products, as well as the frequency of smoking and the additives and flavors that have been added to these products, all impact the level of tobacco dependence^21^. Nicotine boosts the brain’s synthesis of neurotransmitters that is linked to reward (dopamine) within minutes of taking the first puff, which in itself encourages further tobacco use^22^. Along with the rewarding effects of nicotine, several other factors, such as peer pressure, stress, and the environment, encourage people to continue smoking^23^.

Globally, two out of every three smokers try to quit; however, most of these attempts fail due to the withdrawal symptoms that tobacco users may encounter^24^. Withdrawal symptoms occur in all forms of tobacco, both smoked and smokeless tobacco products. It may begin one to two days after quitting and peak within the first week. It includes stress, an increase in appetite, depression, difficulty in concentration, irritability, and sleeping difficulty^25^. However, these withdrawal symptoms typically subside within two to four weeks from the quit date^26^. Assisted quitting, in the form of behavioral counseling and the use of quit medications, is the best practice approach to helping people who smoke on their quitting journey, as it can increase their chances of quitting success fourfold^27^.

### The impacts of tobacco use

*Health impact*

There is no safe level of tobacco exposure since all forms of tobacco use are hazardous^28^. More than 7000 chemicals have been identified in tobacco smoke; hundreds of them are toxic, and around 70 are carcinogenic^29^. Every year, tobacco products kill around 8 million people, 1.3 million of them are non-smokers who are exposed to secondhand smoke, due to diseases attributed to tobacco use and exposure. The diseases attributed to tobacco products include, but are not limited to, cardiovascular diseases, cancers, and childhood developmental problems^1^. In 2004, around one-third (31%) of premature deaths among children were attributed to secondhand smoke exposure^28^.

According to the Global Burden of Disease, 2019 report, tobacco use – which includes smoking, chewing tobacco, and exposure to secondhand smoke – is attributed to 1077 (8.7%) of the total number of deaths in Oman, with a significant sex disparity of 862 (10.9%) deaths for males and 216 (4.8%) deaths for females (Table 2)^30^. Those aged ≥50 years had the highest percentage of tobacco-related mortality, 902 (23.3%), followed by those aged 15–49 years, 170 (4.9%). Additionally, tobacco smoking is responsible for 37636 (4.4%) disability-adjusted life years (DALYs), with people aged ≥50 years being most impacted. Even though some tobacco users may consume tobacco for decades, the health impacts may not become apparent until later in life.

**Table 2:** Disability adjusted life years (DALYs) and mortality attributed to tobacco use by age and sex, Oman 2019

*Age (years)*	*Death* *n (%)*			*DALYs* *n (%)*		
	*Total*	*Male*	*Female*	*Total*	*Male*	*Female*
<15	5 (1.0)	2 (0.7)	3 (1.5)	505 (0.7)	215 (0.6)	290 (0.9)
15–49	170 (4.9)	149 (5.4)	21 (3.0)	12622 (2.6)	11177 (3.4)	1445 (1.0)
≥50	902 (23.3)	711 (31.5)	192 (6.3)	24507 (19.4)	15200 (26.8)	4776 (9.4)
Total	1077 (8.7)	862 (10.9)	216 (4.8)	37636 (4.4)	31125 (5.7)	6511 (2.1)

DALYS: Disability Adjusted Life Years. Source: GBD 2019, Oman^30^.

Table 3 explores diseases attributable to tobacco use (smoked products, smokeless tobacco, and exposure to secondhand smoking) in Oman, according to the Global Burden of Disease (GBD) report 2019^30^. Ischemic heart disease (IHD) accounted for 27.57% of total deaths in Oman, of which 16.77% were attributed to tobacco use. Stroke, on the other hand, is accountable for 8.32% of total deaths, of which 10.34% were attributable to tobacco use. The highest DALY burden attributable to tobacco use was observed in IHD (26.27%), aortic aneurysms (34.54%), and peripheral artery disease (25.28%).

**Table 3:** Diseases attributable to tobacco use (smoked products, smokeless tobacco, and exposure to secondhand smoking), Oman 2019

*Disease*	*Death (%)*		*DALYs (%)*	
	*Total*	*PAF*	*Total*	*PAF*
**Cardiovascular**				
Ischemic heart disease	27.57	16.77	9.71	20.26
Stroke	8.32	10.34	3.61	11.71
Aortic aneurysm	0.23	32.33	0.10	34.54
Atrial fibrillation	0.33	2.42	0.13	5.81
Peripheral artery diseases	0.03	23.81	0.01	25.28
**Cancer**				
Trachea, bronchus, lung	1.16	44.93	0.45	41.99
Colon, rectum	1.08	5.55	0.44	5.26
Stomach	0.84	7.80	0.33	7.03
Esophageal	0.28	22.87	0.03	10.36
Liver cancer	0.67	8.84	0.32	7.96
Leukemia	0.72	8.46	0.47	5.63
Prostate	0.44	3.41	0.14	3.62
Lip, oral cavity	0.17	19.06	0.08	18.01
Pancreatic	0.69	8.37	0.27	8.01
Nasopharynx	0.05	12.02	0.03	10.36
Bladder	0.22	22.75	0.08	23.76
Larynx	0.08	55.42	0.04	52.94
Other pharynx	0.06	32.94	0.03	31.69
Cervical	0.19	4.11	0.09	3.67
Kidney	0.18	8.11	0.09	6.59
**Respiratory**				
COPD	1.63	37.41	0.84	32.02
LRTI	3.29	17.04	1.50	15.97
TB	0.15	12.46	0.11	10.59
Asthma	NA	NA	0.81	4.30
**Metabolic**				
DM	5.70	11.50	3.86	11.99

PAF: population attribution fraction. DALYs: disability adjusted life years. COPD: chronic obstructive pulmonary disease. LRTI: lower respiratory tract infection. TB: tuberculosis. DM: diabetis mellitus. NA: not available. Source GBD 2019, Oman^30^.

Oman has a low cancer death rate, but the death rate attributable to tobacco was highest for larynx cancer (55.42%), trachea, bronchus, and lung cancer (44.93%). On the other hand, those with the highest DALY burden attributed to tobacco were found among patients with larynx, trachea, bronchus, and lung cancer (Table 3).

Over one-third (37.41%) of the deaths from chronic obstructive pulmonary disease (COPD) were attributable to tobacco use. On the other hand, 11.5% of diabetes mellitus deaths were attributable to tobacco use. Overall, the direct and indirect deaths and DALYs attributable to tobacco use in Oman were evident, yet limited tobacco control measures have been established to overcome this emerging epidemic^30^.

*Economic impact*

The economic cost of tobacco consumption is significant globally, with a 2012 study revealing that smoking-related disorders cost the healthcare system $467 billion, or 5.7% of total healthcare expenditure^31^. This cost is almost equivalent to 1.8% of global gross domestic products (GDP), with 40% of it incurred in developing countries^31^.

The health system in Oman comprises public and private sectors; however, the majority depend on the public sector^32^. Healthcare costs per capita have steadily increased in recent decades, and this trend is anticipated to continue. In 2021, total per capita spending was US$1022, with US$889 coming from government health spending^32^. This is expected to increase to US$1329 by 2050, with US$11089 coming from government health spending and just US$155 coming from prepaid private spending. The rise in the prevalence of noncommunicable diseases and its underlying causes, such as tobacco use, could explain the rise in healthcare costs per capita, putting additional strain on the healthcare system.

When considering tobacco control, no health technology assessment has been conducted to assess the direct and indirect costs of tobacco use at the individual or national level in Oman. One study that was conducted in 2016 in the Gulf Cooperation Council countries found that the overall monetary cost of smoking and exposure to secondhand smoke in Oman was US$637 million, representing almost 1% of total GDP. Secondhand smoking expenses account for 22.9% of total GDP expenses, while active smoking costs account for 74.1%^33^. The direct, morbidity, and mortality costs accounted for 45.2%, 22.5%, and 32.5% of the total tobacco cost, respectively^33^. Men and middle-aged people who smoke incur the greatest proportion of indirect costs. Overall, more research is required to better understand the direct and indirect costs of tobacco use on the individual, healthcare system, environment, and society as a whole.

### The benefits of quitting tobacco

The benefits of quitting tobacco can be seen both immediately and in the long-term^34^. It can extend from the individual level to a wider environmental, societal, and healthcare system level. The blood pressure and heart rate are reported to return to normal levels within 30 minutes after quitting. The nicotine level returns to zero in 24 hours post quitting. In the long-term, the risk of having a stroke or developing coronary heart disease is decreased to that of a non-smoker after 15 years. Furthermore, tobacco cessation reduces the risk of developing cancers, including but not limited to lung, oral, pancreatic, stomach, liver, bladder, and colorectal cancers. Even people with existing smoking-related diseases can benefit from quitting.

Globally, the majority of smokers want to quit, and many have attempted to do so; nevertheless, the addictive nature of tobacco products makes it difficult for individuals to quit, forcing them to continue smoking and endure devastating adverse health effects^34^. Brief advice from a healthcare professional can increase the quitting success rate by 30%, while intensive advice, including pharmacological and behavioral support, can increase the chance of quitting by 84%^35^. Quitting smoking can improve overall health, reduce tobacco-related illnesses, and prolong life. The World Bank estimates that the current smoking rates will result in the deaths of 520 million people by 2050. Halving young adults’ consumption will save 20 million lives, whereas halving adult smoking will save 180 million lives^36^. Only adult smoking cessation will significantly reduce tobacco-related deaths in the short- to medium-term. Overall, addressing, preventing, and controlling NCDs, with a focus on predisposing risk factors including tobacco use, should be seen as a national priority in Oman.

### Attitudes toward tobacco cessation

**Figure 3:**
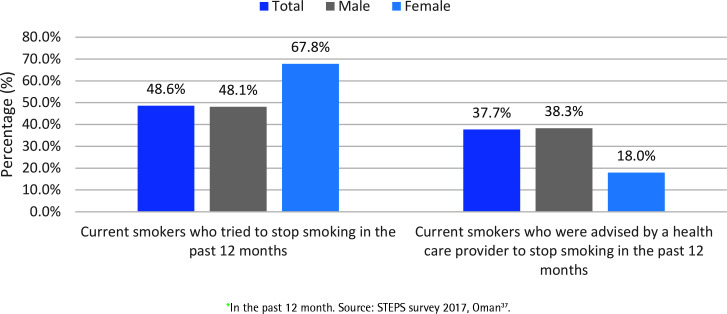
Pattern of tobacco cessation among adults in Oman in the past 12 months based on the STEPS survey 2017, Oman (N=9053)

Globally, 6 in 10 individuals who smoke would like to quit, and 4 in 10 individuals who smoke have attempted to quit in the last 12 months^34^. In Oman, tobacco cessation was reported in the STEPS survey 2017 (Figure 3)^37^. Almost half of male adults (48.2%) and two-thirds of female adults (67.8%) attempted to quit smoking in the 12 months preceding the study. More than one-third of all current smokers (37.7%) were advised to quit smoking by healthcare personnel, with men receiving this advice twice as often as women^37^. There are no available data on the outcome of the quit attempts.

**Figure 4:**
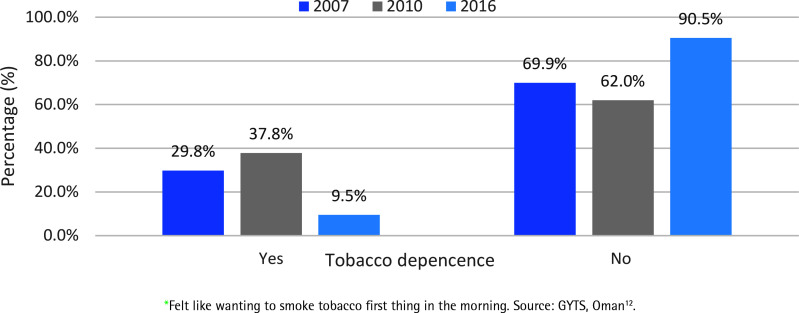
Tobacco dependence* among children based on GYTS 2007 (N=2297), 2010 (N=1620), and 2016 (N=2208)

Tobacco use dependence among youth has been reported in the Global Youth Tobacco Survey^20^. According to the GYTS 2007, and 2010, around one-third of youngsters who smoke demonstrated some degree of dependence (felt like wanting to smoke tobacco first thing in the morning) (Figure 4)^12^. However, according to the 2016 GYTS report, the proportion of tobacco-dependent children has decreased to 10%. The exact reason for this drop is not clear.

### Tobacco cessation service in Oman compared to the Gulf Cooperation Council countries

According to the Framework Convention on Tobacco Control Article 14, countries that ratify the WHO FCTC are mandated to offer tobacco cessation service at its best practice^2^. This can be done by incorporating tobacco cessation service within the nation’s healthcare system^2^. As a best practice approach to assisting people in quitting smoking, the WHO FCTC recommends that each tobacco control program incorporates at least three different forms of cessation treatments. These include pharmacotherapy, cessation counseling at a primary care system, and a toll-free quitline. However, only 23 countries, representing 32% of the world’s population, provide comprehensive cessation programs with full or partial cost coverage to help individuals quit smoking^35^.

In Oman, little progress has been made in building a national tobacco cessation program^9^. When it comes to the minimal WHO requirement of establishing a cessation program, there is no national tobacco cessation service, toll free quitline, or free smoking-cessation medications. The health service is provided in selective primary healthcare settings; however, there are no available data on the quit rate. Brief advice in the form of asking, advising, assessing, assisting, and arranging (the 5As) is not routinely provided at each clinical encounter^27^. Although the healthcare system record (Al Shifa 3+) has a specialized page for tobacco cessation, neither tobacco cessation medications nor a toll-free quitline are available^10^.

When comparing the tobacco cessation service in Oman with that in other Gulf Cooperation Council (GCC) countries, it is clearly demonstrated that Oman has not achieved its obligation to establish a comprehensive tobacco cessation program. Table 4 summarizes the key performance indicators of Article 14 of the WHO FCTC in the GCC countries based on 2022 data. While Saudi Arabia and the United Arab Emirates (UAE) have already implemented the three minimum standards of a comprehensive tobacco control program, as well as providing services in hospitals and community settings, Oman has yet to do so. Several factors could explain the discrepancy in implementing tobacco cessation programs in the GCC countries, including cultural and social norms, economic factors, political will, healthcare infrastructure, and global health trends. Cultural acceptance and sensitivity can influence the priorities of tobacco cessation programs, while economic factors like revenue from tobacco taxes can make governments reluctant to introduce aggressive tobacco control measures. Economic diversification can also influence the willingness of countries to invest in public health initiatives like tobacco cessation programs.

**Table 4:** Progress made to implement Article 14 of framework convention on tobacco control in Gulf Cooperation Council countries, based on WHO 2022 dataset

Indicator	Oman	Bahrain	Kuwait	Qatar	KSA	UAE
Access to toll free Quitline	No	No	Yes	No	Yes	Yes
Smoking cessation support (SCS)^a^ is available in primary care facilities	No	Yes, some^§^	Yes, some^§^	Yes, some^§^	Yes, most^§§^	Yes, some^§^
SCS is available in hospitals	No	Yes, some^§^	No	Yes, some^§^	Yes, some^§^	Yes, some^§^
SCS is available in offices of health professionals^b^	No	No	No	No	Yes, some^§^	Yes, some^§^
SCS is available in the community^c^	No	No	No	No	Yes, most^§§^	Yes, some^§^
SCS is available in other settings^d^	No	No	Yes, some^§^	Yes, some^§^	Yes, some^§^	Yes, some^§^
NRT - legally sold	No	Yes	Yes	Yes	Yes	Yes
NRT- place available	NA	Pharmacy*	Pharmacy*	Pharmacy**	Pharmacy*	Pharmacy*
Bupropion - legally sold	No	Yes	No	Yes	Yes	No
Bupropion - place available	NA	Pharmacy**	NA	Pharmacy**	Pharmacy**	NA
Varenicline - legally sold	No	Yes	Yes	Yes	Yes	Yes
Varenicline - place available	NA	Pharmacy**	Pharmacy**	Pharmacy**	Pharmacy**	Pharmacy**

a Offering brief advice or individual or group activities to aid cessation by primary care providers, but does not include quitlines and pharmaceutical treatments.

b Includes private doctors, nurses, psychologists, and medical assistants.

c Includes but is not limited to places of worship, sports or leisure centers, cultural and arts centers, adult training centers, childcare facilities, traditional meeting areas and the services of traditional or faith healers.

d Settings not elsewhere classified. NRT: nicotine replacement therapy.

§ ‘Some’ means in less than half.

§§ ‘Most’ means more than half.

* Without prescription.

** With prescription.

NA: not available. KSA: Kingdom of Saudi Arabia. UAE: United Arab Emirates.

### Strength, weakness, opportunity and threat (SWOT) analysis of tobacco cessation service in Oman

Table 5 summarize the strength, weakness, opportunity and threat (SWOT) analysis to establish a tobacco cessation service in Oman. There is a subnational coordinating system with multidisciplinary teams in place to address different tobacco control measures, including tobacco cessation services. The tobacco cessation clinic is integrated into the health system (Al Shifa 3+). The clinics are run by trained primary healthcare professionals. In addition, there are population-based interventions in place to help smokers quit, such as increased tobacco taxes, banning smoking in enclosed public spaces, banning advertisements, promotions, and sponsorships, and introducing plain packaging. However, there are weaknesses in the tobacco cessation service. There is no national tobacco cessation program that satisfies the WHO MPOWER minimum standard (toll-free Quitline, behavioral counselling, and medication)^9^. Tobacco cessation services are confined to primary healthcare settings, which frequently face staffing and medication availability issues^7^. Despite ongoing attempts to train doctors and nurses in smoking cessation, there is no clear strategy in place to train personnel from other disciplines or healthcare facilities. Brief advice is not routinely provided at each patient encounter^27^. The healthcare system record (Al Shifa) does not maintain track of each patient’s smoking status. Although healthcare providers continue to offer tobacco cessation advice, smokers are less likely to consider quitting without adequate pharmacological support.

**Table 5:** Strength, weakness, opportunity, and threat (SWOT) analysis of tobacco cessation service in Oman

*Strength*	*Weakness*
Subnational coordination system with a multidisciplinary team Integration of tobacco cessation service into the health system Primary healthcare staff run cessation service Population-based interventions to support smokers to quit: 100% tobacco taxation Ban on smoking in enclosed public spaces Ban on advertising, promotion, and sponsorship Introduction of plain packaging	National level Lack of national coordination for evidence-based training No established national tobacco cessation guidelines Challenges with medication availability Service confined to primary healthcare facilities Healthcare facility level Shortage of trained staff Only doctors from the primary health system running the service Time constraints Individual level Lack of social support Insufficient knowledge of cessation services Inadequate knowledge of the harmful effects of tobacco use
***Opportunity***	***Threat***
Develop accessible online training modules for healthcare workers Collaborate with the private sector for tobacco cessation medication availability Ensure the health record system captures patient tobacco use status Incentivize healthcare personnel to provide tobacco cessation services Expand services to secondary and tertiary hospitals and inaccessible areas Collaborate with educational institutions and workplaces to detect and refer tobacco users to tobacco cessation services	Challenges with tobacco cessation medication availability and supply chain issues Insufficient funding for the service Lack of effective leadership that prioritizes tobacco cessation services in the government agenda Introduction of novel tobacco products as quit aids False claims from tobacco industries about tobacco cessation services and tobacco products

Several opportunities exist to build a comprehensive tobacco cessation program in Oman. These include establishing an online training platform for healthcare professionals at different healthcare settings that will provide them with the knowledge and skills necessary to manage smokers who wish to quit. Furthermore, providing brief quit advice at each clinical encounter can raise public awareness and boost the utilization of the service. Coordination with the private sector to ensure a stop-smoking service is available and that no one is left behind. Additionally, there is a need to incentivize the healthcare personnel who provide the service along with their busy schedules. Finally, collaboration is required with educational institutions and companies to identify and refer tobacco users to a tobacco cessation service.

There are several threats to developing a comprehensive tobacco cessation program in Oman. First, lack of funding is the main threats to the sustainability of the service, especially as policymakers view tobacco use as a personal issue rather than a public health issue with negative health and economic consequences. Second, lack of effective and long-term strategic leadership that prioritizes the quit service into the government agenda. Furthermore, the tobacco industry continues to flood the globe with new innovative products with a suboptimal response from the governments to protect their nations from these deadly products. The tobacco industry's false claims about their products, such as e-cigarettes, being aids to quitting remain a significant threat that must be addressed.

### Recommendations and way forward

The current situation shows that Oman is lagging at regional and international levels in terms of implementing a comprehensive tobacco cessation program. This will reflect itself in increased prevalence and health impact of tobacco use in the short- and long-term. Several recommendations can be made to ensure that Oman bridges the gap and implements a comprehensive tobacco cessation program as part of an effective tobacco control strategy. The recommendations can be addressed at the national, healthcare, and individual levels.

At the national level, there should be a national coordination of tobacco cessation program, with effective leadership, that unite the effort to ensure that service (which includes a toll-free quitline, free tobacco cessation medications, and cessation counseling) is available in primary healthcare settings as a best practice approach. Implementing the service across secondary and tertiary healthcare settings, private healthcare settings, community settings, workplaces, and various educational institutions is essential to ensure that ‘no one is left behind’. Second, clear plans should be in place to increase the training capacity of frontline healthcare professionals, such as medical and paramedical staff, to provide tobacco cessation services. A well-structured database should be put in place to capture the progress in building the human resource capacity. Third, make tobacco cessation medications more widely available and inexpensive, even in stores where tobacco products are sold, with pharmacists providing guided advice on how to use them. Fourth, implementation of a tobacco cessation program should be coupled with implementing and enforcing other tobacco control initiatives, as stipulated in the WHO FCTC. For example, implementing measures to promote a tobacco-free campus in schools, healthcare facilities, and government places, as well as banning smoking in outdoor public areas like beaches, stadiums, playgrounds, and private vehicles, can increase the uptake of tobacco cessation services. Fifth, raise awareness about the negative effects of tobacco smoking by including it in the educational curriculum, particularly in schools and undergraduate medical programs. Sixth, collaboration with partners at national, regional, and international levels is critical for sharing, expanding and strengthening tobacco cessation service. Finally, establish a national database to collect and collate data from all tobacco cessation services and human resources with a plan to utilize the data to improve the services.

At healthcare level, several measures can be taken to strengthen tobacco cessation service. First, ensure a clear protocol to screen for tobacco use at all healthcare settings, as tobacco use is one of the root causes of non-communicable diseases, and targeting it will subsequently reduce the burden of non-communicable diseases in Oman. This can be achieved by implementing brief advice about smoking at each clinical encounter as an essential element of health promotion. Second, ensure implementing tobacco cessation service as a best practice in different healthcare settings to support smokers in their quit journey. Both private and public sectors should be enabled to provide the service. To achieve the greatest reach, the service can be offered in-person or remotely (via a toll-free quitline), depending on patient preference. Additionally, undertake targeted approaches for highly vulnerable groups, e.g. children, young people, pregnant women, and smokers with mental disorders, is essential as these are highly impacted by tobacco use and exposure. Third, addressing the concerns of healthcare workers, such as time constraints, a lack of training, the absence of standard clinical protocol, health system issues, and the availability of resources to conduct the service, is crucial to ensuring that they successfully adopt to the new service. The service can be delivered by both medical and paramedical staff. In some countries, the service is well delivered by paramedical staff, who received adequate training in tobacco cessation service.

At an individual and community level, several measures can be taken to improve health literacy and increase uptake of tobacco cessation services. First, ensure credible information is accessible to the public and involve civil society in all phases of public awareness. Second, create voluntary smoke-free areas in private places that are not covered by tobacco-free legislation to further support the smokers to consider quitting. Third, use artificial intelligence to promote a healthy lifestyle, like the WHO’s Florence 2.0, to enhance the delivery of messages about healthier lifestyle choices and mental health^38^.

According to the Sim Smoke tobacco control simulation 2012 model, implementing a comprehensive tobacco cessation program that integrates best practices, can reduce tobacco prevalence by 3.5% and 9.0% after five and forty years, respectively^7^. It can also avoid 8800 fatalities over a 40-year period. However, comprehensive tobacco cessation programs should be implemented as part of comprehensive tobacco control initiatives that reduce tobacco prevalence and assist tobacco users in their quit journeys. When demand reduction policies, are combined, the prevalence of tobacco use falls by 38% and 48% at the five-year and forty-year scales, respectively. Furthermore, implementing all MPOWER measures can prevent 65000 premature deaths^7^.

## CONCLUSION

Tobacco use in Oman is increasing, leading to health consequences at an individual and healthcare system levels. Despite national priorities to address, prevent, and control NCDs, the healthcare system faces challenges in implementing a comprehensive tobacco cessation program under WHO FCTC Article 14. Implementing tobacco cessation service is cost-effective and should be combined with other FCTC strategies to achieve comprehensive tobacco control. Effective collaboration with local, regional, and international partners is crucial for advancing this initiative. Addressing healthcare workers’ concerns and enabling them to deliver best practices are essential for successful service adaptation. More research is needed to understand the factors that enable or act as barriers to implement tobacco cessation services in Oman.

## Data Availability

Data sharing is not applicable to this article as no new data were created.
